# Attitudes Towards End-of-Life Decisions and the Subjective Concepts of Consciousness: An Empirical Analysis

**DOI:** 10.1371/journal.pone.0031735

**Published:** 2012-02-15

**Authors:** Lorella Lotto, Andrea Manfrinati, Davide Rigoni, Rino Rumiati, Giuseppe Sartori, Niels Birbaumer

**Affiliations:** 1 Department of Developmental Psychology and Socialization, University of Padova, Padova, Italy; 2 Department of General Psychology, University of Padova, Padova, Italy; 3 Institute of Medical Psychology and Behavioral Neurobiology, Eberhard-Karls-University, Tuebingen, Germany; French National Centre for Scientific Research, France

## Abstract

**Background:**

People have fought for their civil rights, primarily the right to live in dignity. At present, the development of technology in medicine and healthcare led to an apparent paradox: many people are fighting for the right to die. This study was aimed at testing whether different moral principles are associated with different attitudes towards end-of-life decisions for patients with a severe brain damage.

**Methodology:**

We focused on the ethical decisions about withdrawing life-sustaining treatments in patients with severe brain damage. 202 undergraduate students at the University of Padova were given one description drawn from four profiles describing different pathological states: the permanent vegetative state, the minimally conscious state, the locked-in syndrome, and the terminal illness. Participants were asked to evaluate *how dead* or *how alive* the patient was, and *how appropriate* it was to satisfy the patient's desire.

**Principal Findings:**

We found that the moral principles in which people believe affect not only people's judgments concerning the appropriateness of the withdrawal of life support, but also the perception of the death status of patients with severe brain injury. In particular, we found that the supporters of the Free Choice (FC) principle perceived the death status of the patients with different pathologies differently: the more people believe in the FC, the more they perceived patients as dead in pathologies where conscious awareness is severely impaired. By contrast, participants who agree with the Sanctity of Life (SL) principle did not show differences across pathologies.

**Conclusions:**

These results may shed light on the complex aspects of moral consensus for supporting or rejecting end-of-life decisions.

## Introduction

Over the past decades, the ethical decisions surrounding end-of-life issues [1], [2] posed more questions than they answered. Although there is a substantial consensus among ethicists and medical-legal experts regarding not considering the withdrawal of life-sustaining treatments as criminal acts when these actions are consistent with patient or proxy decisions [3], cases such as Terri Schiavo in the U.S. or Eluana Englaro in Italy brought the right to die issue to the attention of the public. This gave rise to heated debates [4]–[6] between those who opposed to the removal of life support, saying it was comparable to euthanasia, and those who favored it if this was the patient's will (for a definition of euthanasia and related issues, see [7]). A fundamental aspect of the right-to-die debate is tightly related to the question of what it means to be alive [8]. The answer to this question is neither simple nor unambiguous.

In the present study, we investigated how healthy persons perceive patients with severe brain damage: are these patients considered fully alive? Under what circumstances, if ever, do people agree in withdrawing life support from these patients? Jennett [9], [10] reported that nearly 90% of people considered permanent vegetative state worse than death. More recently, Demertzi et al. [11] updated the end-of-life attitudes towards vegetative state and determined the end-of-life attitudes towards minimal conscious state in a sample of medical and paramedical professionals. In this study, we aimed at measuring just “how alive” patients with severe brain injury are perceived.

Two positions characterize the discussion of withdrawal of life support: One position could be described as the Sanctity of Life (SL) principle, according to which every act or omission causing death (even if it eliminates suffering) would constitute a murder and is contrary to the dignity of a human being, and must be considered morally unacceptable. Human life is sacred because from its beginning it involves the creative action of God. “God alone is the Lord of life from its beginning until its end: no one can under any circumstances claim for himself the right to destroy an innocent human being” [12]. On the other hand, people who tend toward a more liberal position ascribe to each person the right to withhold or withdraw life support, if life supports prolong the process of dying and suffering. In other words, people have the right to decide on their own lives. This position may be referred to as Free Choice (FC) principle.

Our main hypothesis is that people who endorse different moral principles have also a different representation of how *alive* a patient affected by severe brain damage is. SL and FC principles might subtend different concepts of being alive, in which the attribution of consciousness plays a fundamental role. Investigating how people perceive patients in which conscious awareness is lost (or severely impaired) provides key information for the understanding of the conflicting positions on the right-to-die issue.

Consciousness has two main components: wakefulness and awareness [13], [14]. These two components are present in various degrees in patients with severe brain damage. For instance, in both the permanent vegetative state (PVS) and minimally conscious state (MCS), the patient is awake, but they differ in awareness: the former is entirely unaware of self and environment, whereas the latter show limited evidence of awareness of self and environment [15], [16]. The Multi Society Task Force on Vegetative State [17] concluded that 3 months after non-traumatic brain damage and 12 months after a traumatic injury, the condition of vegetative patients may be considered permanent. Given proper medical care (i.e. artificial hydration and nutrition), patients can survive for many years. Minimally conscious patients show minimal but definite behavioral evidence of self or environmental awareness, and emergence from this state is characterized by the ability to communicate or use objects functionally [16]. The prognosis of MCS patients is significantly more favorable than those in PVS, however, some patients may remain in a MCS permanently. In contrast, patients with locked-in syndrome are characterized by awareness of the environment, aphonia or hypophonia, and quadriplegia or quadriparesis. Eye or eyelid movements are the main method of communication [18], [19], but is lost in the completely locked-in state [20]. The error rate in diagnosing and separating PVS from MCS and locked-in syndrome is high, ranging from 20 to 40% errors [21]–[24].

In the present study, we aimed at testing whether different moral principles are associated with different attitudes towards the life status of patients with a severe brain damage and towards the agreement to withdraw life-sustaining treatment in patients who ask for it.

## Methods

### Participants

A total of 202 undergraduate students (162 females, age range: 18–29 years) at the University of Padova participated in the study. Participants were tested in a classroom setting and were informed that their responses would remain anonymous. Oral consent to participate was obtained after written information about the study. The study was conducted according to the Declaration of Helsinki and reviewed and approved by the Ethics Committee of the Department of General Psychology of the University of Padova. Written informed consent was waived by the local Ethics Committee because of the anonymous nature of the collected data. Furthermore, participants had the right to refuse to answer any question and to withdraw from the study at any time.

### Study Design and Hypotheses

All participants were first given a booklet containing the instructions and one description drawn from four profiles describing different pathological states including the permanent vegetative state and the minimally conscious state (PVS and MCS, characterized by different degree of awareness), the locked-in syndrome (LI, in which patients are conscious but paralyzed and voiceless), and a description of a patient with terminal illness (TI, in which the patient is conscious and may refuse medical treatments).

Terminal illness was included in the experimental design as control condition to be compared with PVS, MCS, and LI, given that the second paragraph of article n. 32 of the Italian Constitution recognizes a person's right to refuse treatment, even in cases where withholding therapies may lead to death.

Participants were instructed to carefully read the administered material and to answer the questions regarding the scenario by giving their own opinion. They were told that there were no right or wrong answers, and were asked to respond honestly and only after thorough deliberation.

After they had read the instructions, to limit subjective interpretations of the terms “alive” or “dead”, and in order to set a more “objective” starting point, we provided participants with a clinical definition of brain death [25] before introducing the text that described the patient's condition: *“In legal medicine a person in considered dead when brain death is diagnosed. Brain death is the irreversible end of all brain activity.”*

Participants were then given the scenario according to the condition they were randomly assigned (PVS, MCS, LI, or TI). A full description of the four scenarios can be found in Table S1.

After reading the scenario, participants were asked to evaluate *how dead* or *how alive* (according to the conditions they were randomly assigned) the patient was, using a 11-point scale ranging from 0 to 100. Participants who read the PVS, the MCS, or the LI scenarios were asked to imagine that the patient, prior the brain traumatic incident and in full capacity of willing and understanding, had expressed a desire not to remain in such a condition. Participants who read the LI scenario were also told that this decision was not revoked after the onset of the pathological condition. Participants who read the TI scenario were told that the patient had asked for the interruption of medical treatments, with the exception of pain medications.

Participants were then asked to use the same 11-point scale to evaluate how appropriate it was to satisfy the patient's desire. Finally, participants were asked to express their agreement (using a 11-point scale ranging from 0 to 10) with two moral principles describing two different positions concerning the end-of-life issue. The FC principle emphasized the role of human free choice, ascribing to human beings the right to decide for themselves what happens with their lives: *“Every human being has the right to choose freely regarding his own life and well-being. No one can interfere with the right to choose freely and in full capacity of willing and understanding, even when the person's will is to decline medical treatment that is aimed at keeping him alive”*. The SL principle emphasizes that human beings are more than simple living beings, with transcendent qualities that characterize their existence as a person: *“Human life is sacred and inviolable. No one, under any circumstance, can claim the right to end the life of any human being, including himself”.*

Participants completed the questionnaire by providing information about age, gender, level of education, religion, and political attitude. At the end of the study, they were thanked and fully debriefed.

We expected the SL and FC principles to capture different people's judgments concerning the appropriateness in satisfying the patients' requests when they refused (or had expressed the will to refuse) any form of medical treatment. In particular, we hypothesized that the more people endorse the FC principle, the more they would judge the behavior as appropriate. At the same time, the more people endorse the SL principle, the more they would judge the behavior as inappropriate. While these predictions are quite intuitive because it is generally assumed that moral principles guide people's action and judgments, it is difficult to predict whether people endorsing different principles actually have different perceptions of the life status of patients who are affected by different pathologies. In other words, do people endorsing the FC principle perceive patients in which consciousness is impaired the same way as do people who endorse the SL principle?

## Results

### Death Status Perception

To test whether and how the two moral principles affected the perception of the death status of patients, we ran a multiple regression analysis in which pathology (PVS, MCS, LI, and TI) and moral principles (FC = Statement 1; SL = Statement 2) were entered as predictors. The overall equation was significant: R^2^ = .36; F(5, 196) = 21.68; p<.001. As can be seen in Table 1 (Model 1), pathology and FC principle were significant predictors of death status. In other words, people perceived the four pathologies differently, as shown by the *B*-values indicating that PVS received the highest ratings of death status (planned comparisons using TI as a reference class are also reported in Table 1). Furthermore, the more participants agreed with the FC principle, the more they rated the patient described in the scenario as being dead. By including the interaction between pathology and moral principles increased the predictive power of the model, R^2^ = .41; F(11, 190) = 12.05, p<.001 (Model 2 in Table 1).

**Table 1:** Multiple regression analyses on death status perception as dependent variable.

	*F*	*df*	*p*	*B*	*SE*	*t*	*p*
*MODEL 1* (R^2^ = .36)	21.68	5,196	<.0001				
*Pathology*	32.63	3,196	<.0001				
TI *vs.* LI				32.67	4.90	6.67	<.0001
TI *vs.* MCS				26.07	4.93	5.29	<.0001
TI *vs.* PVS				47.51	4.92	9.65	<.0001
FC	5.58	1,196	.019				
SL	.83	1,196	.364				
*MODEL 2* (R^2^ = .41)	12.05	11,190	<.0001				
*Pathology*	34.17	3,190	<.0001				
TI *vs.* LI				32.25	4.78	6.75	<.0001
TI *vs.* MCS				26.17	4.81	5.44	<.0001
TI *vs.* PVS				47.35	4.78	9.90	<.0001
FC	2.61	1,190	.108				
SL	.54	1,190	.465				
*Pathology* x FC	2.97	3,190	.033				
(TI *vs.* LI) x FC				6.12	3.20	1.91	.057
(TI *vs.* MCS) x FC				7.28	2.79	2.61	.001
(TI *vs.* PVS) x FC				7.24	2.76	2.62	.001
*Pathology* x SL	1.13	3,190	.337				

**Figure 1:**
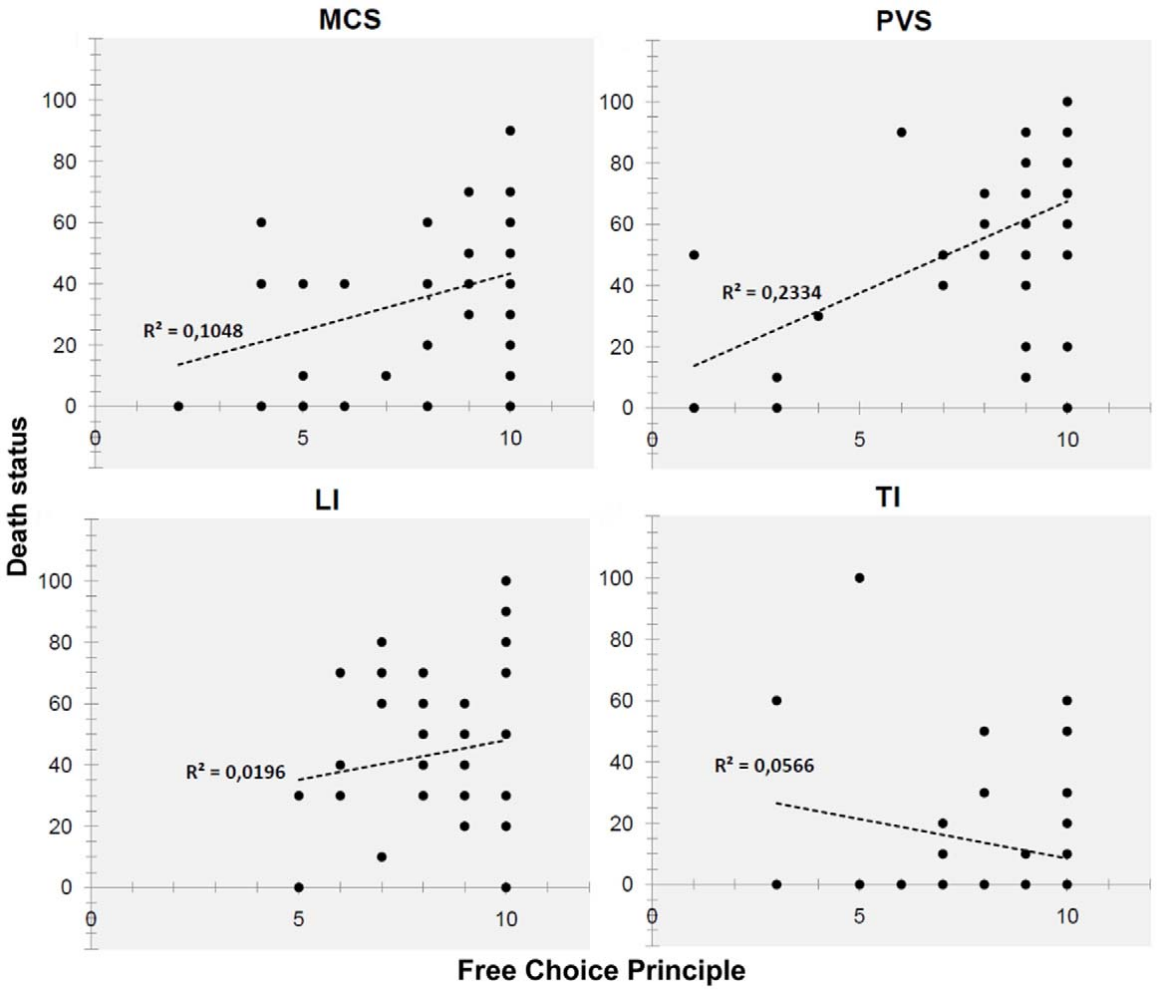
The role of the Free Choice principle in perceiving patients' death status. A regression analysis was run to evaluate how the two moral principles (FC and SL) modulated the evaluation of the death status of patients. The overall equation was significant R^2^ = .41, F(11, 190) = 12.05, p<.0001 and the figure shows the significant interaction between Pathology and FC, where the linear regression curves and their corresponding equations for PVS, MCS, LI, and TI are given.

It is worth noting that in this second regression analysis, pathology was still significant and interacted significantly with the FC principle but not with the SL principle. The significant interaction between pathology and the FC principle indicates that participants endorsing the FC principle perceived the death status of the patients with different pathologies differently: the positive correlation between FC principle and death status hold for MCS and PVS, but not for LI and TI. In particular, when used as a reference for planned comparison, TI was marginally different from LI, but was significantly different from both MCS and PVS (see Table 1). This finding is represented in Figure 1, where the linear regression curves for each of the four pathologies are shown. As it can be seen, the slope of the linear regression curve is steep for MCS and SVP, whereas the curve is much flatter for TI and LI. The lack of interaction between pathology and the SL principle is of particular interest because it suggests that death status ratings provided by participants who endorsed this principle were substantially equal across the four types of pathologies.

### Appropriateness of treatment withdrawal

To test whether and how the two moral principles affected the judgments of appropriateness in satisfying the patient's request to refuse any form of medical treatment, we ran a multiple regression analysis in which FC principle, SL principle, and pathology (PVS, MCS, LI, and TI) were entered as predictors. The overall equation was significant [R^2^ = .65, F(5, 196) = 72.86, p<.0001]. Both the FC and SL principles were significant predictors (F (1, 196) = 219.16, p<.00001 and F (1, 196) = 6.94, p<.01, respectively), showing that participants who highly agreed with the FC principle judged withdrawing of treatment as being highly appropriate, whereas participants who highly agreed with the SL principle expressed lower agreement with this decision. Also pathology reached significance (F (3, 196) = 4.50, p<.01) showing that people judged the four pathologies differently. Planned comparisons using TI as a reference class showed no significant difference for MCS (B = 1.13, SE = 2.91, t = 0.39, p = .70) and marginally significant differences for LI (B = −5.29, SE = 2.93, t = −1.81, p = .07) and PVS (B = 5.43, SE = 2.92, t = 1.86, p = .06, with judgments of appropriateness being lower an higher, respectively, as compared to TI. The interactions between pathology and the FC principle and between pathology and the SL principle did not reach significance when included in the model (p = .60 and p = .09, respectively).

## Discussion

In the present study, we have demonstrated that there is a predictable correlation between the moral principles in which people trust and the agreement to withdraw life-sustaining treatment in patients who ask for it. Specifically, the more people agree with the Free Choice (FC) principle the more they judge treatment withdrawal as appropriate, and the more people agree with the Sanctity of Life (SL) principle the more they judge treatment withdrawal as inappropriate. Furthermore, the lack of significant interactions between pathology and moral principles indicate that appropriateness of treatment withdrawal do not rely on the clinical characteristics defining the different pathological states, but rather on personal beliefs and moral convictions.

What is striking is that the more people believe in the FC principle, the more they perceive patients as dead in instances where conscious awareness is severely impaired or lost (namely, minimally conscious state and permanent vegetative state patients). We view this finding as a useful piece of a puzzle in which psychological and philosophical aspects must be considered in order to better understand end-of-life issues. Indeed, for the supporters of the FC principle, as opposed to those who agree with the SL principle, conscious awareness seems to be central in defining what it does mean to be alive.

The attitude that life is sacred does not necessarily involve religious connotations. Rather, it involves the belief that human life is inviolable and that we have the duty to respect one of the most relevant aspect of the biological life, that is, self-preservation. In this respect, life has an “intrinsic” value that transcends every other characterization in terms of quality of life or social interactions. Our results have shown that this intrinsic value is of great importance for the supporters of the SL principle, as they judged a patient in a permanent vegetative state as much *alive* as other patients affected by different pathologies. On the contrary, for a supporter of the FC principle to be alive is more controversial. Indeed, this concept has been one of the most heavily contested subjects in the philosophical thought. James Rachels [26], for example, claims that we could refer to the term *life* in two different ways. We may refer to living things, that is, things that are *alive*. To be alive means to be a functioning biological organism and, in relation to this, people as well as animals and trees are living things. Conversely, when we speak of life, we may have a different concept of life in mind, a concept that belongs more to biography than to biology. In this respect, a human being is not simply alive; rather, he/she *has a life*, and the permanent loss of conscious awareness could be perceived as similar to the death of the person. Thus, patients in permanent vegetative state might no longer be considered to have a life, despite the fact that they are alive in terms of their basic vital functions.

The role of conscious awareness could be the key concept to understanding the debate surrounding withdrawal decisions. Indeed, a positive correlation between the adherence to the FC principle and death status perception for patients in which conscious awareness is lost (or severely impaired) suggest in this case a sort of “consciousness-life equation”. We might speculate that being aware of both self and the environment (i.e., the content of consciousness [14]) is, for some people, what gives individuals a life. This does not hold for the supporters of the SL principle, for whom there are no differences among the four pathologies. Given this state of affairs, then, we could be induced to think that conflicting viewpoints regarding the ethics of withdrawing or withholding life-sustaining treatments are far from reaching a compromise.

Some limitations of the present study are worth mentioning. First, although both female and male participants were equally distributed in the four pathology conditions, our sample was not balanced for gender and the obtained results might predominantly reflect females' judgments. However, when tested, no significant differences between men and women were found for either death status perception or appropriateness of treatment withdrawal. A further caveat of our study consists of the young age of our sample: attitudes toward life prolongation and disease may change substantially in old age. However, the results obtained by a questionnaire sent to the members of the European Society of Intensive Care Medicine showed that older physicians were more likely than younger physicians to feel that therapy should be withdrawn from patients with no chance of regaining a meaningful life [27]. Furthermore, Blackhall et al. [28] investigated the attitudes of people aged 65 and older toward life sustaining technology demonstrating no significant difference in general attitude for age. Whether the attitudes expressed in our results are translated into real behavior (withdrawal of life support) remains open.

To conclude, the moral principles in which people believe affect not only their judgments concerning the agreement to withdraw life-sustaining treatment in patients with severe brain injury who ask for it, but also the perception of their death status. Indeed, our findings show that in the dispute regarding end-of-life issues there are more than just different values and opinions: How can we deal with different perceptions of the life status of patients?

## Supporting Information

Table S1 **The four scenarios used in the study.**
(DOCX)
