# Supplementary material for: Attitudes Towards End-of-Life Decisions and the Subjective Concepts of Consciousness: An Empirical Analysis
Source: PLoS One. 2012 Feb 15;7(2):e31735. doi: 10.1371/journal.pone.0031735 (PMC3280319; doi:10.1371/journal.pone.0031735)
Supplement: Table S1 — The four scenarios used in the study. (DOCX) [file pone.0031735.s001.docx]

**Table 1. The four scenarios used in the study.**

| **Permanent Vegetative State (PVS)**  *Imagine a patient who suffers from the following pathological conditions:*   - *The patient has been in this state for 5 years following a traumatic brain injury.* - *The patient is unaware of self and environment.* - *The patient’s cortical brain activity is reduced, compared to that of a healthy person.* - *The patient shows some reflexes (e.g. eye movements, yawning, responsiveness to painful stimuli).* - *The patient is not on life-sustaining equipment since cardiocirculatory and respiratory systems are preserved.* - *The patient is unable to swallow and therefore needs artificial feeding.* - *Cognitive functions are not measurable.*     Within the scientific community, it is agreed that PVS patients are awake, but they are assumed to be entirely unaware of self and of their environment. Recently, a few empirical studies demonstrated that, in rare cases, isolated cortical regions are preserved (i.e. are still active) and generate specific responses, such as single word production – although the words are unrelated to the context. It is important to note, however, that the presence of isolated active cortical regions does not predict future recovery in patients with a confirmed diagnosis of PVS. |
| --- |
| **Minimally Conscious State (MCS)**  *Imagine a patient who suffers from the following pathological conditions:*   - *The patient has been in this state for 5 years now following a car accident.* - *The patient is slightly aware of self and environment, and shows gestures and/or verbal responses to simple commands or is capable of object recognition and manipulation.* - *The patient’s cortical brain activity is reduced, compared to that of a healthy person.* - *The patient shows some reflexes (e.g. eye movements, yawning, responsiveness to painful stimuli), and produces recognizable words, although the communication is not always fluent.* - *The patient is not on life-sustaining equipment, since cardiocirculatory and respiratory systems are preserved.* - *The patient is unable to swallow and therefore requires artificial feeding.* - *Cognitive functions are impaired.*   Within the scientific community, it is agreed that MCS patients are aware of self and of the surrounding environment, although awareness is greatly reduced. Some studies suggest that MCS is a transitory state between coma and normal awareness. However, in some cases, this condition can be permanent. |
| **Locked-In Syndrome (LI)**  *Imagine a patient who suffers from the following pathological conditions:*   - *The patient has been in this state for 5 years now following a stroke.* - *The patient is aware of self and environment.* - *The patient’s cortical brain activity is comparable to that of a healthy person.* - *The patient is completely paralyzed (i.e. unable to move, to speak, and cannot show any facial expression) and is only capable of voluntary eye movements. Thus, the patient can only communicate by eye-movements or eye-blinking.* - *Heartbeat is artificially sustained.* - *Respiratory system is artificially sustained.* - *The patient is unable to swallow and therefore requires artificial feeding.* - *Cognitive functions are preserved.*   Within the scientific community, there is agreement that LI patients are entirely aware of self, of their condition, and of the surrounding environment. A number of studies suggest that the cognitive functioning in LI patients is comparable to that of a healthy person even several years after the beginning of the syndrome. |
| **Terminal Illness (TI)**  *Imagine a patient who suffers from the following pathological conditions:*   - *The patient has been in a state of terminal illness for 5 years and cannot be cured or adequately treated.* - *The patient is aware of self and environment.* - *The patient’s cortical brain activity is comparable to that of a healthy person.* - *Motor, perceptual, and communicative skills are comparable to those of a healthy person.* - *Cognitive functions are preserved.* |
